# Acoustic Forceps Based on Focused Acoustic Vortices with Different Topological Charges

**DOI:** 10.3390/s23156874

**Published:** 2023-08-03

**Authors:** Libin Du, Gehao Hu, Yantao Hu, Qingdong Wang

**Affiliations:** 1 College of Ocean Science and Engineering, Shandong University of Science and Technology, Qingdao 266590, China; 2 College of Electronic and Information Engineering, Shandong University of Science and Technology, Qingdao 266590, China; 3 Department of Modern Architecture, Linyi Vocational College, Linyi 276017, China

**Keywords:** focus acoustic vortices, acoustic lens, traps objects, temperature-sensitive drug

## Abstract

For enhanced energy concentration with improved flexibility for object manipulation, a focused acoustic vortex (FAV) is designed using a sector planar piston transducer array and acoustic lens that can produce the effective concentration of the acoustic field to perform the focusing function. Compared to the Gaussian beam, which tends to cause the object to deviate from the axis of acoustic propagation, FAVs can form a central valley region to firmly bind the objects, thus preventing off-target effects. The heat energy in the paraxial region is transferred to the vortex center in the form of heat transfer so that the temperature-sensitive liposomes captured can quickly release drugs, which has a good effect on targeted drug administration. The focused acoustic wave stopped acting on the tissue (gel) for 2 s, the temperature of the vortex center continued to rise, reaching 41.5 °C at the moment of 3.7 s, at which point the liposomes began to release the drug. The FAVs capture the drug and use its thermal effect to achieve accurate and rapid treatment. The simulation results show that the drug release temperature of temperature-sensitive liposomes can be achieved by controlling the action time of the vortices. This study provides a reliable theoretical basis for the clinical application of targeted drugs.

## 1. Introduction

The right focused sound opens up a wide range of therapeutic options. In real-time acoustic or MRI imaging, focused acoustics have been used to identify target locations for the treatment of uterine fibroids, kidney stones, blood clots, excessive bleeding in severe injuries, and cosmetic drugs for fat reduction. To demonstrate that this technique is applicable in many ways to the treatment of neurodegenerative diseases [1], Diane B. Miller analyzed the relevant properties of focused acoustic. At the same time, the wide application of special microvesicles with the same size of red blood cells in targeted therapy has become an important auxiliary means in therapeutic diagnosis. 

Because no one drug type can treat all cancers, multiple chemotherapy drugs need to be packaged to deliver to the affected area, and liposome is a commonly used drug wrapper. Hyperthermia increases blood flow and vascular permeability to the tumor site, thus increasing drug delivery to the tumor site. However, conventional temperature-sensitive liposomes require relatively high temperatures (42–45 °C) to induce drug release, which can cause pain in patients and damage their normal tissues. To find suitable low-temperature-sensitive liposomes with a trigger temperature of 41–42 °C, Hayashi et al. [2] studied temperature-sensitive liposomes composed of various phospholipids. The temperature-sensitive liposomes designed by Kono et al. [3] are composed of dioleylphosphatidyl ethanolamine modified with copolymers of diisopropylacrylamide and *N*-allylpyrrolidine. Paasonen et al. [4] also reported on polymer-coated liposomes with thermally sensitive polys. These temperature-sensitive polymers have functions similar to liposome temperature-controlled drug release. Ultrasound technology can achieve the targeted release of these drugs.

Based on a concave focusing transducer, the energy of the acoustic field can be focused on a certain area to improve energy efficiency, such as HIFU [5,6,7]. When the focusing transducer moves [8], the small bead trapped in the focused field can move in the same direction. To further study the focusing characteristics, an experimental device with micropipette and focusing transducer was set up to quantitatively measure the acoustic trapping force by Hae Gyun Lim [9]. Meanwhile, S. C. Takatori et al., used focused acoustic tweezers to control particles and analyze particle motion under the action of interparticle repulsive force and trapping force [10]. However, larger objects will move away from the focus to the position with lower acoustic intensity, so these acoustic fields cannot achieve accurate control of the objects. 

The acoustic lens focuses on the acoustic beam in another way, which greatly improves the precision of the ball controlled by focusing on the acoustic field. The rapid expansion of phonon crystals [11,12] and acoustic materials [13,14] provide acoustic lenses with a wider application prospect. To obtain the desired acoustic lens, Zigoneanu et al., designed an acoustic metamaterial unit cell whose size can adjust the refractive index of the acoustic lens [15]. Based on a nonlinear acoustic lens consisting of a two-dimensional array of spheres, Donahue et al., controlled the focus by changing the acoustic velocity within the chain by precompressing a single chain [16]. These broke through the shortcomings of traditional acoustic focusing lens geometry and wave curing. The controllable concentration of the vortex field was generated with a sector planar piston transducer array and acoustic lens composition to perform the targeted drug release function.

The unique characteristics of acoustic vortices (AVs) just meet this demand. The AV phase has special helical wavefronts with a null pressure and phase singularity along the central beam axis [17,18]. The angular orbital momentum carried via the vortex field [19] causes the object in the vortex center to rotate. The high acoustic pressure at the vortex radius can be used to trap small objects [20,21] and increase the temperature of diseased tissue. In addition, the capture property of the focused acoustic vortex (FAV) can prevent the off-target phenomenon of the drug and realize the precise drug delivery, and heat transfer in the focal region can meet the drug release requirements of temperature-sensitive liposomes. Arrays of acoustic sources with phase difference are generally used to generate acoustic vortices, such as point acoustic source [22,23,24], circular plane acoustic source [25], sector plane acoustic source [26], and focused acoustic source [27]. The vortices generated by these acoustic sources are becoming larger and larger, which significantly improves the ability to control objects and the utilization rate of acoustic energy.

In this paper, a sector plane piston transducer array and an acoustic lens are used to form FAVs. This flexible and convenient method with low manufacturing cost can not only form vortex fields, but also have a focusing effect and a good thermal effect. Numerical simulation shows that FAVs can bind the ball to the vortex center, and effectively suppress the off-target effect. By analyzing axial and radial temperature distributions and temperature changes over time, the influence of FAVs on hyperthermia was verified. This study is helpful to speed up drug release, improve the accuracy of drug delivery, and provide a more efficient implementation for the clinical use of drugs.

## 2. Methods

**Figure 1:**
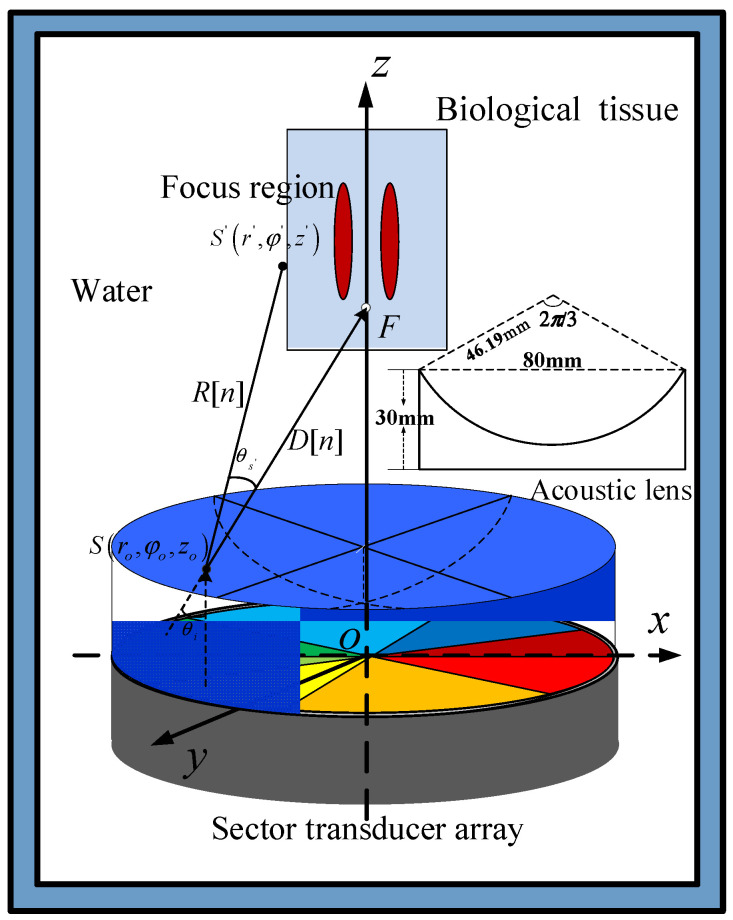
Sketch map of sector transducer array and acoustical lens.

FAV generated by the acoustic lens and planar sector transducer array is schematically shown in Figure 1. *N* planar sector transducers are placed around the center of a circle to form a complete disk whose surface is perpendicular to the *z*-axis. A concave spherical acoustic lens with the same radius is fitted snugly over the transducer array. The phase difference of the drive signals of adjacent units in the array is 2πm/N, where *m* is topological charge (TC). Topological charge *m*, a parameter given in vortex, indicates how many phase twists the sound wave undergoes in a circle. The higher the topological charge is, the faster the wave spins around the axis, and then the greater the angular momentum and acoustic radiation torque generate.

According to the three fundamental equations of small-amplitude acoustic waves, the one-dimensional acoustic pressure wave equation [28] can be described by

(1) $$\frac{1}{c_{0}^{2}}\frac{\partial^{2}p}{\partial t^{2}}=\frac{\partial^{2}p}{\partial x^{2}}$$

where p is the acoustic pressure in the viscous medium, and $c_{0}$ is the acoustic speed of the medium. In the case of simple harmonic waves

(2) $$\frac{\partial^{2}p}{\partial t^{2}}=c_{0}{}^{2}\frac{\partial^{2}p}{\partial x^{2}}$$

where ω is the angular frequency and $k=\frac{\omega }{c}$ is the wave number. Generalized to 3D space, the homogeneous Helmholtz equation satisfied by acoustic pressure is $\nabla^{2}p+k^{2}p=0$. Without considering the nonlinear wave and the shear wave, the wave equation can be further modified as follows

(3) $$\nabla \cdot (\frac{1}{\rho_{c}}\nabla p)+\frac{k^{2}}{\rho_{c}}p=0$$

where ∇⋅ and ∇ are the difference operator and Laplace operator, respectively. $\rho_{c}$ is the density of an ideal medium. Thus, the formula for calculating the acoustic pressure on the surface of the acoustic lens was obtained. Then, taking the surface as the radiation source, the acoustic pressure of the acoustic field in the free space can be calculated by using the principle of ray acoustics.

From the planar sector transducers, acoustic waves travel vertically before reflecting on the concave acoustic lens surface. This satisfies $\cos\left(\theta_{l}\right)=\sqrt{1-\beta^{2}\left(1-\cos\theta_{i}{}^{2}\right)}$, where *θ_i_* and *θ_l_* are the angles of incidence and reflection, respectively, and β is the index of reflection. As shown in Figure 1, an infinite number of points divided from the surface of a planar transducer vibrates with different amplitudes and phases in the radial direction, forming numerous tiny pulsating point sources on the surface of the acoustic lens. Thus, acoustic radiation can be regarded as the sum of radiation from the point source on the surface of the acoustic lens. The distance from the observation point $S^{\prime }\left(r^{\prime },\varphi^{\prime },z^{\prime }\right)$ to the point source $S\left(r_{o},\varphi_{o},z_{o}\right)$ is

(4) $$R\left[n\right]=\sqrt{{\left(r_{o}\cos\varphi_{o}-r^{\prime }\cos\varphi^{\prime }\right)}^{2}+{\left(r_{o}\sin\varphi_{o}-r^{\prime }\sin\varphi^{\prime }\right)}^{2}+{\left(z_{o}-z^{\prime }\right)}^{2}}$$

When an acoustic lens transmits acoustic waves parallel to the *z*-axis to the surface of the lens, the acoustic attenuation of $A(n)=10^{\frac{\alpha *R\left[n\right]}{20}}$, where α is the attenuation coefficient. Thus, the acoustic pressure at the observation position produced by *S* can be calculated with

(5) $$\begin{array}{cc} p^{\prime }\left(r^{\prime },\varphi^{\prime },z^{\prime }\right) & =\sum_{n=1}^{N}\kappa \left[n\right]A\left[n\right]F\left[n\right]p_{n}\left(r_{o},\varphi_{o},z_{o}\right)\\ & =\sum_{n=1}^{N}\frac{\cos\left(\theta_{s^{\prime }}\right)+\cos\left(\theta_{l}\right)}{2}10^{\frac{\alpha *R\left[n\right]}{20}}\frac{e^{-jkR\left[n\right]}}{R\left[n\right]}p_{n}\left(r_{o},\varphi_{o},z_{o}\right) \end{array}$$

where $\kappa (n)=\frac{\cos\left(\theta_{s^{\prime }}\right)+\cos\left(\theta_{l}\right)}{2}$, and $p_{n}\left(r_{o},\varphi_{o},z_{o}\right)$ is transmitted as a point source. $D\left[n\right]=\left[\begin{array}{l} r_{F}\cos\varphi_{F}-r_{o}\cos\varphi_{o}\\ r_{F}\sin\varphi_{F}-r_{o}\sin\varphi_{o}\\ z_{F}-z_{o} \end{array}\right]$ and $D^{\prime }\left[n\right]=\left[\begin{array}{l} r_{o}\cos\varphi_{o}-r^{\prime }\cos\varphi^{\prime }\\ r_{o}\sin\varphi_{o}-r^{\prime }\sin\varphi^{\prime }\\ z_{o}-z^{\prime } \end{array}\right]$ are the direction vector from *S* to focus $F\left(r_{{}_{F}},\varphi_{{}_{F}},z_{F}\right)$ and the direction vector from *S* to *S*’. Thus, the diffraction angle $\theta_{S^{\prime }}$ and incidence angle $\theta_{i}$ can be calculated with $\theta_{S^{\prime }}\mathrm{arcos}=\frac{\langle D\left[n\right],D^{\prime }\left[n\right]\rangle }{\left|D\left[n\right]\right|•\left|D^{\prime }\left[n\right]\right|}$ and $\theta_{i}ar\cos=\frac{\langle D\left[n\right],D\left[z\right]\rangle }{\left|D\left[n\right]\right|•\left|D\left[z\right]\right|}$, where $D[z]=\left[\begin{array}{l} r_{F}\cos\varphi_{F}\\ r_{F}\sin\varphi_{F}\\ z_{F} \end{array}\right]$ is the *z*-axis direction vector.

Assuming that solid particles are stationary in the time mean field, the time average acoustic radiant force (AGF) and torques [29,30] can be described with

(6) $$F=\int_{\Gamma }\langle \left(\sigma_{2}-\rho_{0}\langle \left(n•v_{1}\right)v_{1}\rangle \right)\rangle d\Gamma$$

(7) $$T=\int_{\Gamma }r\times \langle \left(\sigma_{2}-\rho_{0}\langle \left(n•v_{1}\right)v_{1}\rangle \right)\rangle d\Gamma$$

where 〈 〉 is the time operator, n is the normal outward vector on the surface of the particle Γ**,** and **r** is a position vector pointing from the center of mass to a point on the surface of a particle. Thus, the second order radiation pressure tensor can be calculated with

(8) $$\langle \sigma_{2}\rangle =-\langle p_{2}\rangle I=-\left(\frac{\langle p_{1}{}^{2}\rangle }{2\rho_{0}c_{0}{}^{2}}-\frac{\rho_{0}\langle {\left|v_{1}\right|}^{2}\rangle }{2}\right)I$$

where I is the identity matrix, $p_{2}$ is the second order acoustic pressure and $\left|v_{1}\right|$ is the amplitude of the first order vibration velocity. Approximated as a plane wave, the acoustic intensity of ultrasonic propagation in the focal region is $I=\langle p^{2}\rangle {/(\rho }_{0}c_{0})$. Considering the amplitude of complex harmonics $C_{n}$, the acoustic intensity is normalized to $\tilde{I}(R,Z)=4\sum_{n=1}^{\infty }{\left|C_{n}\right|}^{2}$, where $\tilde{I}=I/I_{0}=I/(p_{0}{}^{2}{/2\rho }_{0}c_{0})$ is the time average acoustic intensity. 

The ultrasonic heat source equation is derived below. The temperature of biological tissue in the focal area rises rapidly due to the thermal effect of ultrasound. The heat energy Q absorbed by biological tissues satisfies

(9) $$Q=4p_{0}{}^{2}\sum_{n=1}^{\infty }\alpha_{n}{\left|C_{n}\right|}^{2}/(\rho_{0}c_{0})$$

which was derived from the second order radiation pressure tensor

(10) $$\langle \sigma_{2}\rangle =-\langle p_{2}\rangle I=-\left(\frac{\langle p_{1}{}^{2}\rangle }{2\rho_{0}c_{0}{}^{2}}-\frac{\rho_{0}\langle {\left|v_{1}\right|}^{2}\rangle }{2}\right)I$$

and the spatial gradient of sound intensity [31] (Q=−∇⋅I) which represents the heat absorption of tissue per unit volume per unit time.

Because it is difficult to distinguish the absorption and scattering region of ultrasonic waves, the attenuation coefficient is usually equivalent to the absorption coefficient $\alpha_{n}$ [32] in biological tissue. Thus, the heat source Q can be described by

(11) $$Q=4p_{0}{}^{2}\sum_{n=1}^{\infty }\alpha {\left|C_{n}\right|}^{2}/(\rho_{0}c_{0})$$

In the process of hyperthermia, the temperature of biological tissue keeps rising and diffusing, and the temperature change of the tissue can be described using

(12) $$\rho_{0}C_{t}\frac{\partial T}{\partial t}=\kappa_{T}\nabla^{2}T+Q$$

Here, $C_{t}$ and $\kappa_{T}$ are the specific heat and heat transfer coefficient, respectively, T is the real-time temperature of the biological tissue.

The energy of the planar transducer array’s sound field is scattered, but the energy of the FAVs is primarily focused in the area along the lateral axis, which is more favorable for the impact of vortices on particles. The thermal effect of FAVs is discussed for the first time. The vortex center of FAV can hold the drug particles firmly, and the focused acoustic heat can cause the liposomes to be released quickly. The FAVs could also be used as an efficient means to improve the therapeutic effect of treatments with the accumulated drug particles, thereby enabling more potential applications in clinical practice.

## 3. Numerical Simulations and Experimental Studies

### 3.1. Transducer and Acoustic lens

Numerical simulations of FAVs were carried out using MATLAB. The parameters of the sector transducer element in numerical studies were set for radius *a* = 40 mm, *N* = 8, and Δφ=π/4. The phase differences of the drive signals for the transducer array were set to Δϕ=2πm/8 for generating AVs. The lens parameters were set for transmission velocity *c* = 2146 m/s, acoustic lens radius of curvature *F* = 46.19 mm, diameter 2*a* = 80 mm, height *h* = 30 mm, density $\rho =1023\;\mathrm{kg}/m^{3}$, attenuation coefficient α=1.8dB/cm, and refractive index β = 1.56 at a frequency of 500 kHz.

### 3.2. Focused Acoustic Fields

**Figure 2:**
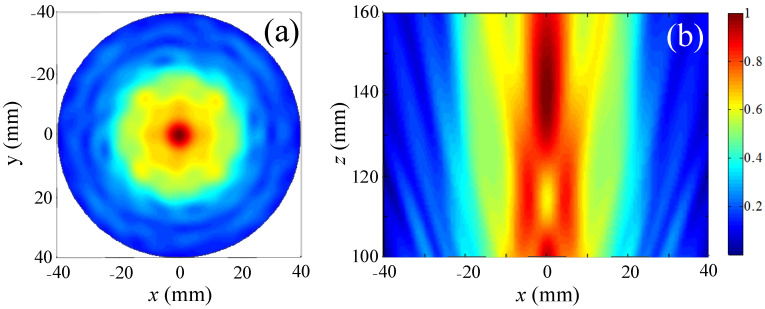
Distribution of acoustic pressure for topological charges *m* = 0, (**a**) cross-sectional at *z* = 140 mm and (**b**) axial profile.

The focused acoustic field generated by the composite system was first simulated with *m* = 0 and the corresponding normalized cross-sectional distance *z* = 140 mm was obtained as plotted in Figure 2a with the spherical acoustic lens center positioned at the origin. This shows a focal region with a radius of 20 mm formed along the *z*-axis. The axial profile pressure distributions were also calculated as illustrated in Figure 2b. This shows that the high pressure region is formed at *z* = 130–150 mm and a potential well is formed at *z* = 116 mm. It is clear that the focus has the highest pressure, making it impossible to collect microscopic particles there.

### 3.3. Focused Acoustic Vortex Fields

**Figure 3:**
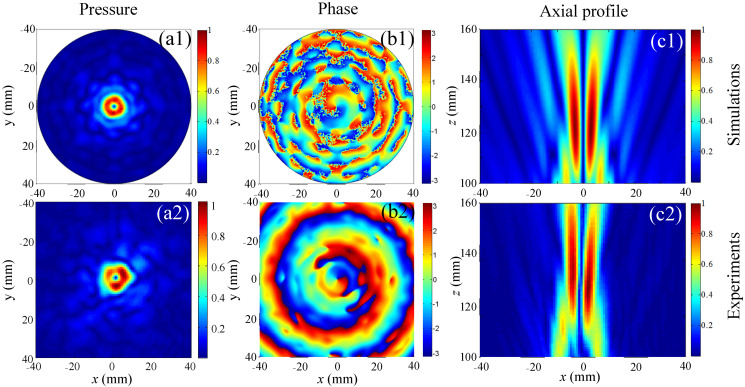
Cross-sectional distributions of (**column 1**) pressure and (**column 2**) phase at z = 118 mm, and (**column 3**) the axial pressure profiles for simulations (**line 1**) and experimental measurements (**line 2**).

Based on the above parameters, the cross-sectional and axial profile pressure distributions of the vortex fields were numerically calculated as plotted and shown Figure 3a1,b1,c1. The acoustic pressure on the cross-section forms a circular distribution at *z* = 118 mm, and the vortex radius is about 3.4 mm. Although the acoustic energy is dispersed 7 mm away from the propagation axis, a potential well with zero pressure will be formed at any axial position between 110 mm and 160 mm with its radius positively correlated with the axial distance. Thus, for particles smaller than the wavelength, FAVs with stronger AGFs can capture particles in the vortex center and rotate them. 

The acoustic fields of the FAVs with *m* = 1 were measured with the scanning measurement apparatus with a motion step of 0.5 mm. The axial and cross-sectional distributions of pressure and phase shown in Figure 3(a2,b2,c2) strongly agree with the calculation results. The agreement between the axial profiles in Figure 3(c2) and the calculation in Figure 3(c1) attests to the good acoustic focusing produced by the acoustic lens. The annular pressure distributions with clear phase spirals in Figure 3(b2) strongly agree with the numerical results in Figure 3(b1). The FAVs are demonstrated via the clear annular pressure distributions and phase spirals around the vortex centers of a pressure null. The phase variation from 0 to 2π around the vortex center agrees exactly with the corresponding topological charge *m* = 1.

### 3.4. Acoustic Gradient Forces

**Figure 4:**
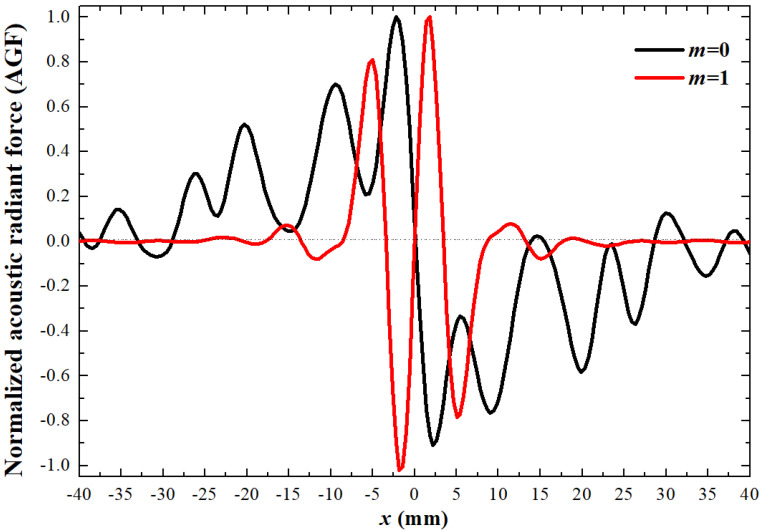
The normalized AGF for topological charges *m* = 0 and *m* = 1, axial distance *z* = 130 mm.

To verify that particles are affected by acoustic waves in the acoustic field, the radial AGF distributions of acoustic fields with *m* = 1 and 0 are plotted in Figure 4, assuming that the elastic object to be manipulated is a spherical polyethylene particle with *ρ_p_* = 918 kg/m^3^, *c_p_* = 1900 m/s, and *a_p_* = 0.1 mm. The black and red curves are the normalized AGF distributions with *m* = 0 and 1, respectively. There are obvious radial AGF fluctuations with positive and negative values representing AGF in the positive and negative directions, respectively. As shown in Figure 4, when *m* = 0, the particle is easily pushed away from the acoustic propagation axis, causing the particle to move radially. However, when *m* = 1, the polyethylene particle is firmly bound to the propagation axis (vortex center) by the AGF, which effectively inhibits the generation of off-target effect. FAVs with strengthened AGF can form a central valley region to firmly bind the drugs in the location of lesion, thus preventing off-target effects. The heat energy in the paraxial region is transferred to the vortex center in the form of heat transfer so that the temperature-sensitive liposomes captured can quickly release drugs, which has a good effect on targeted drug administration. This indicates that FAVs have more advantages than planar transducers in the control of drug particles, especially the temperature-sensitive release of drug particles. 

### 3.5. Temperature Fields of FAVs 

**Figure 5:**
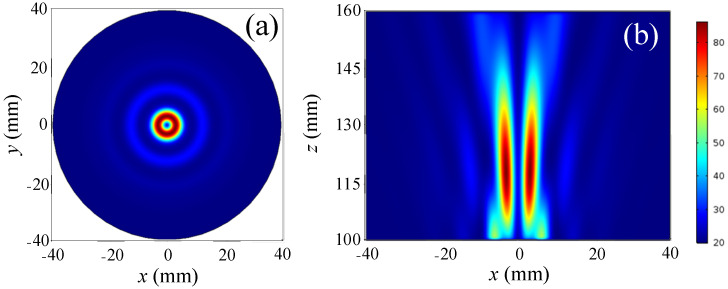
Numerical simulations: (**a**) cross-section at *z* = 118 mm and (**b**) axial profile temperature distributions in the focal region at time *t* = 2 s with topological charges *m* = 1.

However, the application of FAVs to nanoscale drug particles has not been reported. We have demonstrated the control effect of poly-FAVs on the particle, cross-section, and axial profile distribution of temperature fields, when FAVs act on tissue-mimicking (gel) for 2 s as shown in Figure 5a,b, respectively. With a low-temperature vortex center and a high-temperature annular region, the temperature distribution of the cross-section is similar to the pressure, which provides great advantages for the realization of local heat treatment. Unlike the distribution of acoustic pressure fields, the focal region of temperature fields as shown in Figure 5b is closer to the acoustic source than that of acoustic pressure fields.

### 3.6. Temperature Distributions of the Focal Region 

**Figure 6:**
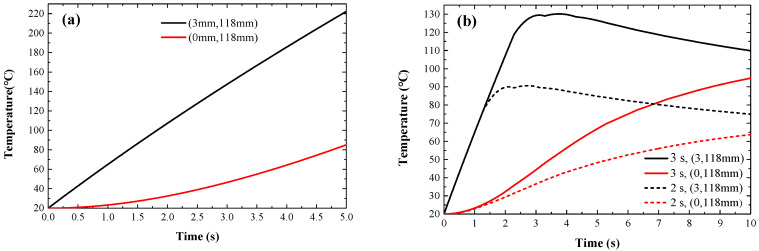
Temperature distributions of two points (0, 118 mm) and (3 mm, 118 mm) in the focal region with time: (**a**) 5 s corresponds to the action of the acoustic source and (**b**) 2 s (dashed line) and 3 s (solid line) correspond to the action of the acoustic source.

The temperature-sensitive liposome is trapped in the vortex center after being captured by the FAVs. The thermal effect of the FAVs increases the temperature of the liposome and the liposome releases the internal drug when the temperature approaches 41–42 °C. To study the temperature characteristics over time in the temperature fields, the temperature distributions of the vortex center (0, 118 mm) and focal point (3 mm, 118 mm) over time were obtained, as shown in Figure 6a. This shows that, over time, the temperature at the vortex center gradually increases faster (red curve) and the temperature at the focal point increases almost linearly within 5 s. These theoretical results provide a valuable theoretical basis for controlling the temperature of the vortex center through the conduction of heat energy. To study the effect of the action time on temperature, the rule of temperature variation in the vortex center and focal point after 2 s (dashed line) and 3 s (solid line) of action is shown in Figure 6b marked by red and black lines, respectively. After the vortex stopped acting at 2 s and 3 s, the temperature obviously began to drop after reaching 90 °C and 107 °C as a result of thermal diffusion. However, the temperature in the vortex center continued to rise, reaching 41.5 °C at 3.7 s and 2.8 s, respectively. The favorable result provides a feasible method to achieve temperature-sensitive liposomes for drug release by controlling the action time of the vortex.

## 4. Results

A sector planar piston transducer array with an acoustic lens was used to form FAVs for the application of a new targeted therapy. The distributions of acoustic pressure, temperature, and AGF were theoretically analyzed and corresponding simulation results were given, respectively. It was found that there is a good agreement between theoretical research and numerical simulation. FAV has a good focusing effect, but the acoustic lens can concentrate most of the energy near the axis of acoustic propagation. The potential well of FAVs could firmly trap drug particles, which can effectively prevent off-target effects in the non-vortex-focused acoustic field. The radial distribution curve of the AGF shows that the AGF on the drug particles always points towards the vortex center, while the AGF of non-vortex propagation is opposite. A small area of high temperature is conducive to the realization of local heat treatment, as shown on the temperature distributions. The temperature of the annular region is high, while the temperature of the vortex center is low. The drug was released at the vortex center when the temperature of the thermosensitive liposome reached 41.5 °C via the effect of thermal diffusion.

## 5. Conclusions

By installing a concave spherical acoustic lens on a circular array of planar sector transducers, an FAV was designed to enhance the utilization of acoustic energy with improved flexibility and the ability to trap drug particles. Based on the acoustic refraction of a concave spherical acoustic lens, a theoretical model of the FAV was established. Theoretical analysis and numerical study proved that this study is helpful to speed up the release of drugs, improve the accuracy of drug delivery, and provide a more efficient implementation scheme for the clinical use of drugs.

## Data Availability

Not applicable.

## References

[1] Miller D., O’Callaghan J. (2017). New horizons for focused ultrasound (FUS)-therapeutic applications in neurodegenerative diseases. Metab. Clin. Exp..

[2] Hayashi H., Kono K., Takagishi T. (1998). Temperature-dependent associating property of liposomes modified with a thermosensitive polymer. Bioconjug Chem..

[3] Kono K., Yoshino K., Takagishi T. (2002). Effect of poly (ethylene glycol) grafts on temperature-sensitivity of thermosensitive polymermodified liposomes. J. Control. Release.

[4] Paasonen L., Romberg B., Storm G., Yliperttula M., Urtti A., Hennink W. (2007). Temperature-sensitive poly (N-(2-hydroxypropyl) methacrylamide mono/dilactate)-coated liposomes for triggered contents release. Bioconjug Chem..

[5] Hutchinson L. (2011). HIFU is effective for unresectable HCC. Nat. Rev. Clin. Oncol..

[6] Kennedy J. (2005). High-intensity focused ultrasound in the treatment of solid tumours. Nat. Rev. Cancer.

[7] Gavrilov L. (2013). High Intensity Focused Ultrasound in Medicine. J. Acoust. Soc. Am..

[8] Lee J., Teh S., Lee A., Kim H., Lee C., Shung K. (2009). Single beam acoustic trapping. Appl. Phys. Lett..

[9] Lim H., Li Y., Lin M., Yoon C., Lee C., Jung H., Shung K. (2016). Calibration of Trapping Force on Cell-Size Objects from Ultrahigh-Frequency Single-Beam Acoustic Tweezer. IEEE Trans. Ultrason. Ferroelectr. Freq. Control.

[10] Takatori S., Dier R., Vermant J. (2016). Acoustic trapping of active matter. Nat. Commun..

[11] Liu Z., Zhang X., Mao Y., Zhu Y., Yang Z., Chan C., Sheng P. (2000). Locally resonant sonic materials. Science.

[12] Lu J., Qiu C., Ke M., Liu Z. (2016). Valley Vortex States in Sonic Crystals. Phys. Rev. Lett..

[13] Liang Z., Li J. (2012). Extreme acoustic metamaterial by coiling up space. Phys. Rev. Lett..

[14] Cheng Y., Zhou C., Yuan B., Wu D., Wei Q., Liu X. (2015). Ultra-sparse metasurface for high reflection of low-frequency acoustic based on artificial Mie resonances. Nat. Mater..

[15] Zigoneanu L., Popa B., Cummer S. (2011). Design and measurements of a broadband two-dimensional acoustic lens. Phys. Rev. B.

[16] Donahue C., Anzel P., Bonanomi L. (2014). Experimental realization of a nonlinear acoustic lens with a tunable focus. Appl. Phys. Lett..

[17] Thomas J., Marchiano R. (2003). Pseudo Angular Momentum and Topological Charge Conservation for Nonlinear Acoustical Vortices. Phys. Rev. Lett..

[18] Bliokh K., Freilikher V. (2006). Polarization transport of transverse acoustic waves: Berry phase and spin Hall effect of phonons. Phys. Rev. B.

[19] Allen L., Beijersbergen M., Spreeuw R., Woerdman J. (1992). Orbital angular momentum of light and the transformation of Laguerre-Gaussian laser modes. Phys. Rev. A.

[20] Skeldon K., Wilson C., Edgar M., Padgett M. (2008). An acoustic spanner and its associated rotational Doppler shift. New J. Phys..

[21] Kang S., Yeh C. (2010). Potential-Well Model in Acoustic Tweezers. IEEE Trans. Ultrason. Ferr..

[22] Yang L., Ma Q., Tu J., Zhang D. (2013). Phase-coded approach for controllable generation of acoustical vortices. J. Appl. Phys..

[23] Zheng H., Gao L., Ma Q., Zhang D. (2014). Pressure distribution based optimization of phase-coded acoustical vortices. J. Appl. Phys..

[24] Gao L., Zheng H., Ma Q., Tu J., Zhang D. (2014). Linear phase distribution of acoustical vortices. J. Appl. Phys..

[25] Li Y., Guo G., Ma Q., Tu J., Zhang D. (2017). Deep-level stereoscopic multiple traps of acoustic vortices. J. Appl. Phys..

[26] Wang Q., Li Y., Ma Q., Guo G., Tu J., Zhang D. (2018). Near-field multiple traps of paraxial acoustic vortices with strengthened, gradient force generated by sector transducer array. J. Appl. Phys..

[27] Baresch D., Thomas J., Marchiano R. (2013). Spherical vortex beams of high radial degree for enhanced single-beam tweezers. J. Appl. Phys..

[28] Cheng J. (2012). Fundamentals of Acoustics.

[29] Jürg D., Hahn P., Leibacher I., Möller D., Schwarz T., Wang J. (2012). Acoustofluidics 19: Ultrasonic microrobotics in cavities: Devices and numerical simulation. Lab Chip.

[30] Wijaya F., Lim K.M. (2016). Numerical calculation of acoustic radiation force and torque on non-spherical particles in Bessel beams. J. Acoust. Soc. Am..

[31] Bailey M., Khokhlova V., Sapozhnikov O. (2003). Physical mechanisms of the therapeutic effect of ultraacoustic (a review). Acoust. Phys..

[32] Pennes H. (1948). Analysis of tissue and arterial blood temperatures in the resting human forearm. J. Appl. Physiol..

